# Association between *fok1* polymorphism of vitamin D receptor gene with uterine leiomyoma in Turkish populations

**DOI:** 10.4274/jtgga.2018.0002

**Published:** 2018-08-06

**Authors:** Seda Güleç Yılmaz, Tuğçe Gül, Rukset Attar, Gazi Yıldırım, Turgay İşbir

**Affiliations:** 1 Department of Molecular Medicine, Yeditepe University, Institute of Health Sciences, İstanbul, Turkey; 2 Departments of Obstetrics and Gynecology, Yeditepe University School of Medicine, İstanbul, Turkey; 3 Department of Medical Biology, Yeditepe University School of Medicine, İstanbul, Turkey

**Keywords:** Vitamin D receptor, polymorphism, fok1, uterine leiomyoma

## Abstract

**Objective:**

The aim of this research was to determine the association between the *fok1* polymorphism and uterine leiomyomas.

**Material and Methods:**

For genotyping the *fok1* polymorphism of the vitamin D receptor, real-time polymerase chain reaction was performed on blood samples of uterine leiomyoma (n=27) and control (n=33) groups. For statistical analyses, SPSS v.23 software (SPSS Inc., Chicago, IL, USA) was used.

**Results:**

A statistically significant difference was observed for the frequency of the CC genotype between the uterine leiomyoma and control groups, and the frequencies of the T allele in the uterine leiomyoma groups were significantly higher than in the control group.

**Conclusion:**

The presence of the *fok1* CC genotype may be a risk-reducing factor and the T allele may be a potential risk factor for developing uterine leiomyoma.

## Introduction

Uterine leiomyomas are the most common benign muscle tumors (1,2,3,4), with up to 60% prevalence in women of a fertile age (1). Leiomyomas can be present in multiples, round, and can vary in size from several millimeters to 20 cm or more (2). They are clonal tumors arising from the smooth muscle cells of the uterus and contain excessive extracellular components (3). In addition, their mitotic activity is usually low (2). It is known that the pathogenesis of uterine leiomyoma is multifactorial, although not exactly elucidated (4). In patients with uterine leiomyomas, dysmenorrhea, pelvic pain, menorrhagia, infertility, complications during pregnancy (1), heavy menstrual bleeding and bladder discomfort can be observed (5). Uterine leiomyomas decrease the quality of life because of these adverse effects.

Vitamin D is a hormone that regulates some biologic processes in normal tissues, such as cellular differentiation and proliferation. It also regulates proliferation, apoptosis, and cell adhesion in cancer tissues (6). The vitamin D precursor, 7-dehydrocholesterol, can be synthesized by the human liver. When 7-dehydrocholesterol is activated by ultraviolet light from sun, 1,25-dihydroxycholecalciferol is synthesized, which is the active form of vitamin D that shows its effects via the human vitamin D receptor (VDR) gene (7). 

The VDR is known to be a member of the steroid hormone receptor superfamily of nuclear receptors (8), and it interacts with VDR response elements (VDRE) (6). The gene encoding the VDR is located on human chromosome 12q13-12q14 (8), and it has five promoter regions and 14 exons (eight of them protein coding and six of them non-protein coding) (9). When vitamin D binds to the VDR, it contacts with the retinoic acid receptor, as well. After the complex is formed, it binds to VDRE, and its transcription is regulated (10). At least 196 VDR single nucleotide polymorphisms have been identified (9). The *fok1* polymorphism was detected on the first ATG codon of the VDR gene (11). 

Mun et al. (12) hypothesized an association between the *fok1* polymorphism of the VDR gene and female reproductive cancers in their meta-analysis. These studies consisted of 4107 ovarian and 16.453 breast cancer cases, which examined risks with fixed or random effects models under heterozygous, homozygous, dominant, and recessive models. It was shown that the *fok1* polymorphism was related to increased risk, whereas the BsmI polymorphism was associated with a decreased risk for gynecologic cancers (12). In addition to the meta-analysis, some significant associations between the *fok1* polymorphism and prostate, colorectal, skin, gastric, melanoma, and non-small cell lung cancers have been discovered (6). Vitamin D levels of females have been associated with uterine leiomyomas (13). Paffoni et al. (16) noticed that the vitamin D levels of patients with uterine leiomyomas were significantly lower than in control groups. Uterine leiomyoma is also associated with obesity. Sex steroids (e.g., estrogen) are responsible for regulating adipocyte metabolism (14). Thus, uterine leiomyomas can be considered as an estrogen-dependent tumors (1). Marshall et al. (15) and Paffoni et al. (16) studied the association between body mass index (BMI) and uterine leiomyomas. 

The aim of this research was to determine the association between the *fok1* polymorphism and uterine leiomyomas. A part of the pathogenesis of uterine leiomyomas is thought to be related to the metabolism of vitamin D, which was illuminated through the determination of an association between the *fok1* polymorphism and uterine leiomyomas.

## Material and Methods

For this study, samples were collected from the gynecology and obstetrics departments of Yeditepe and İstanbul University, to form control (n=33) and uterine leiomyoma groups (n=27). To compare and detect any association between the *fok1* polymorphism and the presence of a uterine leiomyoma, the first group of patients was formed according to the presence of uterine leiomyoma. The second group was made according to the BMI ranges in order to study the association between BMI, uterine leiomyoma, and the *fok1* polymorphism. The BMI ranges were determined according to the classification of the World Health Organization. 

Peripheral blood samples were collected into EDTA-containing tubes with the consent of the volunteer participants in this research. DNA isolation was performed using an iPrep Purification Instrument (Invitrogen, Life Technologies, Carlsbad, California, USA) with 350 µL of peripheral blood and an Invitrogen iPrep PureLink gDNA blood isolation kit (Invitrogen, Life Technologies, Carlsbad, California, USA). After isolation, the DNA samples were measured spectrophotometrically by NanoDrop 2000 (Thermo Scientific, Waltham, Massachusetts, USA) for genotyping. An Applied Biosystems 7500 Fast Real-Time PCR instrument (Applied Biosystems, Foster City, CA, USA) was used for genotyping the VDR gene *fok1* polymorphism through the use of a TaqMan Genotyping Assay, TaqMan Genotyping Master Mix and 100 ng DNA of sample. With these materials, the reaction mixture was prepared as recommended by the manufacturer, and then 10 µL of the reaction mixture and 1 µL of the sample DNA were added to each well in the plate for genotyping. The polymerase chain reaction (PCR) reaction conditions consisted of a 10 minute at 95 °C hold stage followed by 40 cycles of 15 seconds at 92 °C for denaturation, and 60 seconds at 60 °C for extension. Allelic discriminations of the samples were detected by collecting and interpreting the fluorescent signals of the hybridization probes using the software associated with the Applied Biosystems 7500 Fast Real-Time PCR instrument. 

For statistical analyses, SPSS v.23 software (SPSS Inc., Chicago, IL, USA) was used. Student’s t-test was used to compare the means of the ages and the BMI ranges of the control and uterine leiomyoma groups. Chi-square and Fisher’s exact tests were performed by detecting the significant differences between the genotypes of the groups. Risk estimations were examined through binary logistic regression analysis as an odds ratio (OR) with a 95% confidence interval (CI). When the p value of the statistical test was lower than 0.05, the test was considered to be statistically significant.

## Results

The demographic characteristics of the uterine leiomyoma group (n=27) and the control group (n=33) are given in Table 1. The mean ages of the patients with uterine leiomyoma and the control group were 38.19±10.217 and 39.76±11.771 years, respectively. As a result of Student’s t test, there was no statistical significance between the mean ages of the groups (p=0.587). Additionally, the mean BMI of the patients with uterine leiomyomas and the control group were 24.44 and 22.13 kg/m2, respectively. The mean BMI of the uterine leiomyoma group was significantly higher than the control group (p=0.034*).

The allelic and genotypic frequencies for *fok1* (rs2228570) were analyzed. As a result of this analysis, the frequencies of the CC, CT, and TT genotypes among the patients with uterine leiomyoma were 40.7%, 48.1%, and 11.1%, respectively. The frequencies of the CC, CT, and TT genotypes among the people in the control group were 66.7%, 30.3%, and 3.0%. When the statistical tests were performed, statistical significance was observed for the frequency of CC genotype occurrence between the uterine leiomyoma and control groups [χ2=4.033, p=0.045*, OR: 2.91, 95% CI: (0.120-0.987)], and no significance was found for the other genotypes. The frequency of the T allele in the uterine leiomyoma group was significantly higher than in the control group [χ2=4.033, p=0.045*, OR: 2.909, 95% CI: (1.013-8.355)] (Table 2). 

When the BMI ranges of patients with uterine leiomyomas versus the control group were analyzed, it was observed that there was no statistical significance.

## Discussion

Uterine leiomyoma is the most common benign tumor in women, and because of its adverse effects, uterine leiomyomas decrease quality of the life (1,2,3,4). In the United States of America, about $34.4 billion per year is spent for the treatment of uterine leiomyomas (17); however, in Turkey, this number could be lower because epidemiologically, uterine leiomyomas are more common in African-American women than in Caucasians (15,17,18).

Vitamin D has roles in proliferation, apoptosis, and cell adhesion in cancer tissues showing its effects through the VDR (6,7). Al-Hendy et al. (19) showed that vitamin D induces nuclear VDR. At least 196 VDR single nucleotide polymorphisms have been identified (9). The *fok1* polymorphism was detected on the first ATG code of the VDR gene (11). Mun et al. (12) hypothesized an association between the *fok1* polymorphism of the VDR gene and female reproductive cancers in their meta-analysis (12). VDR polymorphisms have not only been associated with uterine leiomyomas. Some studies found the *fok1* polymorphism associated with breast, ovarian, adenomatous polypopsis, colon (7), non-small cell lung, prostate, gastric, and melanoma cancers (6). Monitoring VDR gene polymorphisms and its molecular associations could give a route for the prognosis of uterine leiomyomas, and further, identify potential targets for the development of novel treatments as personlised medicine (12).

Bläuer et al. (20), compared the differences in effect of vitamin D on leiomyoma and myometrial tissues, and determined that vitamin D inhibited the proliferation of tissues *in vitro* (20). An article written by Sharan et al. (21), who used human uterine leiomyoma cells supported this result.

Shahbazi (22) wrote an original article about the *fok1* polymorphism and uterine leiomyoma. To the best of our knowledge, that report was the first and ours is the second on the association between the *fok1* polymorphism and uterine leiomyomas, and the results were similar in both studies, yet they conducted on different populations. Also, the present study was performed using RT-PCR with high-tech apparatus. From our analysis, the frequencies of CC, CT, and TT genotypes among patients with uterine leiomyoma were 40.7%, 48.1%, and 11.1%, respectively. The frequencies of CC, CT, and TT genotypes in the control group were 66.7%, 30.3%, and 3.0%. According to Shahbazi’s research, the frequencies of CC, CT and TT genotypes among the patients with uterine leiomyoma were 35.5%, 42.5% and 22%, respectively, and the frequencies of these genotypes in the control group were 51%, 32%, and 17%. Shahbazi determined that the *fok1* polymorphism could impact the development of leiomyomas (22).

In conclusion, we found a statistically significant difference in the mean BMI of the uterine leiomyoma group versus the controls (p=0.034*). Carrying the CC genotype seemed to significantly decrease the risk of developing a uterine leiomyoma (p=0.045*), and carrying the T allele significantly increased the risk of developing a uterine leiomyoma (p=0.045*) in the Turkish population. These results, however, are limited by the small sample size and require additional studies on a larger number of patients for verification.

## Figures and Tables

**Table 1:**
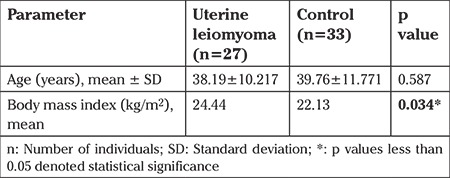
Demographic information of the study groups

**Table 2:**
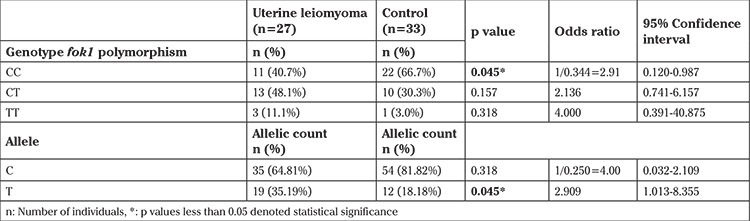
VDR *fok1* (rs2228570) genotype and allele frequencies of the groups
